# Analysis of the role of acetylation in *Giardia lamblia* and the giardicidal potential of garcinol

**DOI:** 10.3389/fmicb.2024.1513053

**Published:** 2025-01-03

**Authors:** Rocío G. Patolsky, Jerónimo Laiolo, Luciano Díaz-Pérez, Gabriel Luna Pizarro, Gonzalo F. Mayol, María C. Touz, Constanza Feliziani, Andrea S. Rópolo

**Affiliations:** ^1^Instituto de Investigación Médica Mercedes y Martín Ferreyra, INIMEC – Consejo Nacional de Investigaciones Científicas y Técnicas (CONICET), Universidad Nacional de Córdoba, Córdoba, Argentina; ^2^Universidad Católica de Córdoba, Córdoba, Argentina

**Keywords:** acetylation, parasites, antigiardial drugs, lysine acetyltransferases, garcinol

## Abstract

**Introduction:**

Post-translational modifications of proteins provide cellular physiology with a broad range of adaptability to the external environment flexibly and rapidly. In the case of the protozoan parasite *Giardia lamblia*, the study of these modifications has gained relevance in recent years, mainly focusing on methylation and deacetylation of proteins. This study investigates the significance of acetylation in this protozoan parasite.

**Methods:**

This study explores the role of acetylation in *G. lamblia* through a combination of immunofluorescence assays, manipulation of acetyltransferase enzymes, and the use of garcinol, an acetylation inhibitor, during the growth phase.

**Results:**

The acetylation of histone marks H3K9 and H3K27 occurs during growth and is followed by deacetylation during encystation. Transfections modifying acetyltransferase activity induced a latent cellular state, underscoring the importance of protein acetylation for parasite survival. Garcinol treatment during growth caused significant morphological changes, including plasma membrane blebbing and apoptotic-like bodies. Immunofluorescence revealed these bodies contained α-tubulin/acetylated α-tubulin and reactive oxygen species. Flow cytometry and Annexin V staining indicated early apoptosis within 24 hours of treatment. Additionally, garcinol led to the deacetylation of H3K9 and H3K27, with redistribution of tubulin and acetylated tubulin from microtubules to the cytosol. Significantly, garcinol prevented parasite recrudescence after treatment withdrawal.

**Discussion:**

These results demonstrate that acetylation is essential for trophozoite survival and highlight the natural histone acetyltransferase inhibitor garcinol as a potential candidate for drug development against giardiasis, considering its giardicidal activity.

## 1 Introduction

Living organisms continuously interact with their environment and other organisms throughout their existence. Parasitism, a form of association between species, involves one benefiting at the expense of the other. *Giardia lamblia* (syn: *G. intestinalis*, *G. duodenalis*), an intestinal protozoan parasite, survives and differentiates in a potentially hostile environment, parasitizing the small intestine of humans and other vertebrates, causing giardiasis (Adam, 2001, 2021). Symptoms range from asymptomatic to diarrhea, abdominal distension, nausea, and, in some cases, a chronic condition that can significantly impact the quality of life. This potential for chronic conditions underscores the need for effective prevention and treatment strategies. Globally, *G. lamblia* is the third most common agent of diarrheal disease in children under five, with over 200 million people worldwide experiencing diarrhea associated with giardiasis annually (Fakhri et al., 2021). In Argentina, giardiasis cases have been reported in 15 out of 23 provinces, with a prevalence in the human population varying significantly from 3.4 to 64.8%, depending on clinical status, sociodemographic conditions, and geographic area (Rivero et al., 2020).

Like most unicellular parasites, *G. lamblia* displays easily distinguishable morphological life stages. This microorganism showcases vegetative and replicating activity with high metabolic rates. These stages are known as trophozoites and are located within the host’s intestinal tract. Trophozoites have a pear-like shape, two transcriptionally active nuclei, a cytoskeleton, a ventral disk, and four pairs of flagella for movement. Conversely, when they transition to their encapsulated form with reduced metabolic activity, they are referred to as cysts. *G. lamblia’s* bidirectional life cycle between these two stages, facilitated by various cellular differentiation processes, is a fascinating aspect of its biology. As these changes need to occur quickly and efficiently, epigenetics emerges as a facilitator of this process. Within this broad field, abundant posttranslational modifications of histones have been identified in eukaryotic cells, particularly at the exposed N-terminal ends that are not part of the octamer. These modifications help control access to DNA, regulating chromatin structure and function. The transcriptional effects of histone modifications arise from either the direct biophysical impact of the modification or through the catalytic activity of proteins and complexes that recognize and target the residues [reviewed in Bannister and Kouzarides (2011) and Csizmok and Forman-Kay (2018)].

Among the various residues that can be modified in histones, lysine is unique because it undergoes multiple modifications, such as acetylation, methylation, ubiquitination, SUMOylation, and hydroxylation. These modifications are mutually exclusive for a lysine residue at the same position in a protein, providing an antagonistic regulation mechanism for different lysine modifications. Histone acetylation decreases the net positive charge on the nucleosome, destabilizing the association of histones with negatively charged DNA, enabling more accessible access to chromatin-associated factors, and enhancing transcription. Therefore, maintaining a balance in the levels and activity of enzymes that add or remove acetyl groups from histones can either activate or repress transcription (Bannister and Kouzarides, 2011; Shvedunova and Akhtar, 2022).

Initially, enzymes that add acetyl groups to proteins were called HATs (histone acetyltransferases). Still, as early as 2007, a systematic nomenclature based on the acronym KAT (lysine acetyltransferase) was suggested (Allis et al., 2007). Moreover, while many human proteins contain acetyl-lysine, only a few of these enzymes have been identified, raising the question of how such a limited number of enzymes maintain such a vast acetylome. Molecular characterization reveals that some enzymes are versatile and target multiple protein substrates, not just histones. KATs in higher organisms are categorized into three families: the GNAT family, the p300/CBP group, and the MYST proteins (Drazic et al., 2016). Despite the various types of KATs, many of them share the same catalytic site; for instance, the CREB-binding protein (CBP) and p300 facilitate the acetylation of histones H3Lys18 (H3K18) and H3K27, while GCN5 and p300/CBP-associated factor (PCAF) promote the acetylation of H3K9 (Jin et al., 2011).

In the genome of *Giardia lamblia*, canonical histones are represented: two copies of H2A, H2B, and H3 and three copies of H4.^1^ There are two variants, H3B and CenH3, and an absence of H1 (Wu et al., 2000; Dawson et al., 2007). Until a few years ago, nothing in *Giardia* was known about histone modifications or the enzymes involved in this process. However, in 2021, the first map of post-translational histone modifications obtained by mass spectrometry was published, and some changes were validated using commercial antibodies (Carranza et al., 2016; Emery-Corbin et al., 2021).

Regarding histone deacetylation, there are intriguing results concerning the role of KDAC enzymes in the encystation process. Sonda et al. (2010) demonstrated that inhibiting classical KDACs (GL50803_3281) with FR235222 suppresses cyst production by downregulating specific encystation genes (Sonda et al., 2010). They also noted a decrease in acetylation levels during encystation, indicating the necessity of histone deacetylation for this process. Furthermore, Carranza et al. identified NAD + -dependent KDACs from the sirtuin family that are involved in the early stages of encystation, suggesting their role as metabolic sensors (Carranza et al., 2016). These researchers proposed that the high parasitic density in the intestine during infection could lead to nutrient scarcity, pH alterations, and increased NAD+ levels, activating sirtuins. This activation would result in the deacetylation of specific residues like H4K8ac and H4K16ac, which exhibit reduced acetylation levels in the initial phases of encystation.

Regarding KDAC inhibitors, it has been reported that Trichostatin A, tubastatin, and nicotinamide inhibit the proliferation of trophozoites, but the mechanism of action was not studied in depth (Sonda et al., 2010; Carranza et al., 2016; Lagunas-Rangel et al., 2020). More recently, it has been described that another KDAC inhibitor, called KH-TFMDI, disrupts cell growth, causing cytokinesis arrest and the formation of vacuoles with autophagosome-like characteristics (Gadelha et al., 2019; Oliveira et al., 2022). Therefore, the authors consider this compound a promising drug for treating giardiasis.

In this work, we explore the flip side of deacetylation, which is the acetylation of lysines in *G. lamblia*. Through various approaches, we analyze the significance of this modification in the life cycle of *Giardia* and postulate garcinol, a histone acetyltransferase inhibitor, as a potential therapeutic agent for treating giardiasis.

## 2 Materials and methods

### 2.1 *Giardia lamblia* culture

Trophozoites of the WB isolate, clone 1267, were obtained from ATCC (American Type Culture Collection catalog number 50582). Cells were cultured in a 16 mL screw-cap tube using TYI-S33 medium supplemented with 10% adult bovine serum and bovine bile and incubated at 37°C (Diamond et al., 1978). Growing trophozoites form a monolayer in the tube, which can be recovered by placing the tubes at 4°C for 30 min, followed by centrifugation at 2,500 rpm for 15 min. The *in vitro* encystation process was carried out in two stages. In the first stage, the growth medium of the trophozoites cultured to confluence for 24 h was discarded, and the tubes containing trophozoites adhered to their walls were filled with pre-encystation medium (complete growth medium without bovine bile). Finally, this medium was replaced by an encystation medium containing a concentration of 0.45% porcine bile and 0.01% lactic acid, with a pH of 7.8 (Gillin et al., 1989). This medium change induces the differentiation of trophozoites into cysts, which lose their adherence capacity and remain free in the encystation medium.

### 2.2 *Giardia* transgenic cell lines and vectors

Wild type trophozoites (WB/1267) were used as hosts for the expression of transgenic genes and as controls. To constitutively express MYST1, MYST2, and GCN5, the plasmid pTubHApac with the tag HA at the C-terminus was employed (Touz et al., 2003). The vector contains a puromycin cassette controlled by the endogenous non-regulated tubulin promoter for cell selection. The tubulin promoter was replaced by a sequence containing the putative endogenous promoter to express the enzymes under their own promoter. Moreover, to inhibit the enzymes’ expression, ORF sequences were amplified and ligated to the pTub-HApac in the opposite direction, resulting in the antisense vector. The primers used are detailed in Table 1. Stable trophozoite transfection was performed as was previously described (Yee and Nash, 1995; Singer et al., 1998). Drug-resistant trophozoites were usually apparent by 7–10 days post-transfection.

**Table 1:** Primers sequences.

	5′–3′ sequence
Overexpression	
GCN5-FW	CATTGGGCCCTCAGACGGTGCTTCCTCCCCACAGA
GCN5-RV	CATTGATATCGAAACCAAGGTCGGCAAAGGCCTGTTCTA
MYST1-FW	CATTGGGCCCCGTTTTTGTCCCCCAGAGGCTCTGT
MYST1-RV	CATTGATATCTACTATTCTGTCTCTGCGCCTGTACATC
MYST2-FW	CATTGGGCCCACAGCAAAAGATCTTACTGAGCCGT
MYST2-RV	CATTGATATCTTTGTACTCGTAACACATGTTGACATATG
Endogenous promoter	
GCN5-eng-fw	CATTTCTAGACCTAGAACGCGAAACGATCTTTGTC
MYST1-eng-fw	CATTTCTAGAAGCCATCTTGAAGGTGGTGCAGATT
MYST2-edg-fw	CATTTCTAGATTCCGTTAACAGTGCCTGTTCAATG
GCN5-eng-rv	CATTGGGCCCCATGTTGTTTTTTGGTTAACTGGCA
MYST1-eng-rv	CATTGGGCCCCATTTCATTTAATGTTCAAAGGAAC
MYST2-edg-rv	CATTGGGCCCCATGGGTACAATGAAACGATCTTTT
Antisense	
GCN5-AS-FW	CATTGATATCATGTCAGACGGTGCTTCCTCCCCAC
GCN5-AS-RV	CATTGGGCCCATCAGAAGCATCCCCATTAAAGGTC
MYST1-AS-FW	CATTGATATCATGCGTTTTTGTCCCCCAGAGGCTC
MYST1-AS-RV	CATTGGGCCCTGAACTAAGTGTGGCCATCGAGCCA
MYST2-AS-FW	CATTGATATCATGACAGCAAAAGATCTTACTGAGC
MYST2-AS-RV	CATTGGGCCCAACCAGCACTTCGCAAATTCGGCGC

### 2.3 Immunofluorescence assays

Primary antibodies against H3K9ac (1:200; Active Motif, Inc.), H3K27ac (1:200; cell signaling technology), α-Tubulin (1:500; Sigma-Aldrich Co.), and α-Tubulin Acetylated (1:500; Sigma-Aldrich Co.) were used. Cells were fixed with 4% formaldehyde in 1X PBS for 40 min at room temperature, blocked with 3% Bovine Serum Albumin (Sigma-Aldrich Co., USA) and 0.05% Tween (Sigma-Aldrich Co., USA) in 1X PBS. Cells were then incubated with the primary antibody diluted in 1.5% Bovine Serum Albumin (Sigma-Aldrich Co., USA) and 0.05% Tween (Sigma-Aldrich Co., USA) in 1X PBS. After 3 washes with 0.05% Tween in 1X PBS, cells were incubated with Alexa Fluor 488-conjugated secondary antibodies against rabbit or mouse (dilution 1:500, Life Technology) diluted in 1.5% Bovine Serum Albumin (Sigma-Aldrich Co., USA) and 0.05% Tween (Sigma-Aldrich Co., USA) in 1X PBS. Incubation conditions were 1 h at 37°C in a humid chamber. Subsequently, cells were washed three times with 1X PBS and incubated with DAPI (dilution 1:750) for 5 min. Samples were mounted with FluorSave (Merck Group, Darmstadt, Germany) and observed using an Olympus FV1200 confocal microscope. Image analysis was performed using Fiji software^2^ and Adobe Photoshop 8.0 (Adobe System).

### 2.4 Reagents and drugs

The HAT inhibitor garcinol was purchased from Enzo Life Science (USA). Stock solutions of the compound (10 mM) were prepared in dimethyl sulfoxide (DMSO) (Sigma Aldrich Co, St. Louis, USA) and stored at −20°C.

### 2.5 MTT assay

To assess the giardicidal activity of the compound, we performed the MTT assay to measure cell viability by forming formazan crystals due to MTT reduction by viable cells. The compound was dissolved in DMSO (0.5% v/v) to achieve a final concentration of 50 μM. We exposed the drug in a 96-well plate with 5 × 10^5^ cells per well in a final volume of 300 μL of culture medium. Incubation took place for 48 h in a CO_2_ chamber at 37°C. After incubation, the plate was centrifuged at 2,000 rpm for 10 min. We performed three washes by centrifugation and the addition of sterile PBS. Trophozoites were resuspended in 200 μL of PBS, and 20 μL of MTT (5 mg/mL in PBS) was added to each well. Incubation was carried out for 4 h at 37°C. The plate was then centrifuged at 2,000 rpm for 10 min. The supernatant was discarded, and the formazan crystals were solubilized in 100 μL of DMSO. Absorbance was measured using a model 680 microplate reader (Bio-Rad, USA) at 570 nm. The percentage of cytotoxicity was determined by comparing with DMSO-treated control cells, which were considered 100% viable as they exhibited behavior like untreated cells. Additionally, we analyzed a range of decreasing concentrations, with a maximum concentration of 50 μM, to determine the half-maximal inhibitory concentration (IC_50_) using non-linear regression with GraphPad Prism 9.0 software (GraphPad Software, Inc., CA, USA).

### 2.6 Analysis of ultrastructure using transmission electron microscopy (TEM)

10 × 10^6^ cells were grown in a 96-well plate for 48 h at 37°C, where the trophozoites were exposed to the IC_50_ of the compound and a double concentration. DMSO was used as a control. After the incubation, the trophozoites were washed and transferred with cold 1X PBS to a 1.5 mL Eppendorf tube. They were centrifuged at 2,500 rpm for 15 min at 4°C, and the supernatant was discarded. The cells were fixed with a solution containing 4% (v/v) formaldehyde and 2% (v/v) glutaraldehyde in a 0.1 M cacodylate buffer and stored at 4°C. Subsequently, the fixed cells were centrifuged, and the resulting pellets were washed and treated with 1% OsO4 for 1 h. Following dehydration with a graded series of cold acetones, the cells were embedded in Spur resin. Ultrathin sections of 90 nm were prepared after 48 h of polymerization at 60°C using an RMC Power Tome – XL ultramicrotome. Subsequently, the ultrastructural changes were examined using a Hitachi HT 7800 electron microscope (Hitachi, Tokyo, Japan).

### 2.7 Assessment of apoptosis by annexin V/PI double staining assay

To perform this experiment, 5 × 10^4^ trophozoites, suspended in a complete growth medium, were seeded in a 96-well plate. The trophozoites were exposed to the IC_50_ of the compound and a double concentration of it. Control groups included untreated cells as negative controls and cells treated with metronidazole (MTZ) as positive controls. After a 24 h incubation, apoptotic/necrotic cells were processed using the Dead Cell Apoptosis Kit (Thermo-Fisher Scientific, USA) with Annexin V-Alexa Fluor 488 and propidium iodide (PI), following the manufacturer’s instructions. The cells were centrifuged, washed thrice with 1X PBS, and resuspended in 100 μL of 1X Annexin binding buffer. Subsequently, they were incubated with Annexin V-Alexa 488 and PI for 15 min at room temperature in the dark. Flow cytometry was analyzed using the FACSCanto II instrument (Becton & Dickinson, New Jersey, NY, USA). Annexin V/PI dot plot analysis, divided into quadrants, aided in categorizing cells as live (Annexin V−/PI−), early apoptotic (Annexin V+/PI−), late or secondary apoptotic (Annexin V+/PI+), and necrotic (Annexin V−/PI+).

### 2.8 TUNEL assay

Cells, both treated and untreated, were detached by incubation in an ice-water bath for 30 min. They were then collected via centrifugation at 2,500 rpm for 10 min at 4°C and washed twice with 1X PBS. The parasites were resuspended in 1 mL of PBS containing 1% v/v and a drop of the suspension was placed on coverslips pre-coated with poly-L-lysine. The coverslips were incubated at 37°C for 30 min to facilitate trophozoite adhesion. Subsequently, the cells were fixed with 4% v/v formaldehyde in PBS for 40 min, followed by two PBS washes of 5 min each. Permeabilization was performed using 0.1% v/v Triton X-100 for 30 min, after which the cells were washed twice again with PBS for 5 min. Finally, the TUNEL (Terminal deoxynucleotidyl transferase dUTP Nick-End Labeling) assay was performed using the *In Situ* Cell Death Detection Kit, TMR red (Roche), according to the manufacturer’s instructions.

### 2.9 Detection of reactive oxygen species (ROS)

The concentration of reactive oxygen species (ROS) produced by cultured trophozoites (5 × 10^5^ cells) with and without treatment in 96-well plates at 48 h was examined using the Image-iT LIVE Green Reactive Oxygen Species Detection Kit (Invitrogen, MA, USA). The assay is based on a nonfluorescent and cell permeable 5-(and-6)-carboxy-2′,7′-dichlorodihydrofluorescein diacetate (carboxy-H2DCFDA). The carboxy-H2DCFDA permeates the live cells and is deacetylated by intracellular esterases. The reduced fluorescein compound is oxidized by the cellular ROS and emits bright green fluorescence with excitation/ emission maxima of 495/529 nm. In the assay, after cells treatment, they were collected by centrifugation, washed three times with sterile 1X PBS, and resuspended in 100 μL of Working Buffer (25 μM). After incubating for 30 min at 37°C in the dark, additional washes were carried out with sterile 1X PBS, and the final pellet was resuspended in 100 μL of sterile 1X PBS. The fluorescence intensity was measured using the FACS Canto II flow cytometer (Becton & Dickinson, New Jersey, NY, USA). The data was analyzed using FlowJo software. Furthermore, ROS formation was visualized using confocal microscopy Olympus FV1200 with Hoechst as a nuclear marker, incubating for 5 min at 37°C in the dark. The images were processed using the Fiji Image program.

### 2.10 Assessment of *Giardia lamblia* recovery post-treatment with garcinol

Trophozoites (5 × 10^5^ cells) were treated with garcinol in 96-well plates at IC_50_ and at IC_90_ concentration, inhibiting ~50% and ~90% of growth within 48 h at 37°C, respectively. Following treatment, plates were centrifuged and washed with PBS. One plate was immediately subjected to an MTT assay to determine initial viability. The second plate was placed in an anaerobic chamber at 37°C for 48 h to allow any surviving parasites to recover without further treatment. Post-recovery, parasite viability was assessed using the MTT assay. DMSO-treated cells were used as control. Each experiment was performed in triplicate. Cytotoxicity percentages were calculated and analyzed using GraphPad Prism software version 9.0. Statistical significance between viability before and after the 48 h recovery period was determined using a t-test.

### 2.11 Statistics

Results were analyzed for statistical significance using an unpaired, two-sided Student’s t-test with GraphPad Prism 5 Data Analysis Software (GraphPad Software Inc., La Jolla, CA, USA). The mean and standard error of the mean (SEM) were calculated from at least three biologically and technically independent experiments. A *p*-value of less than 0.05 was considered significant and is indicated by asterisks in the figures.

## 3 Results

### 3.1 Localization and changes on histone acetylation during growth and encystation

**Figure 1:**
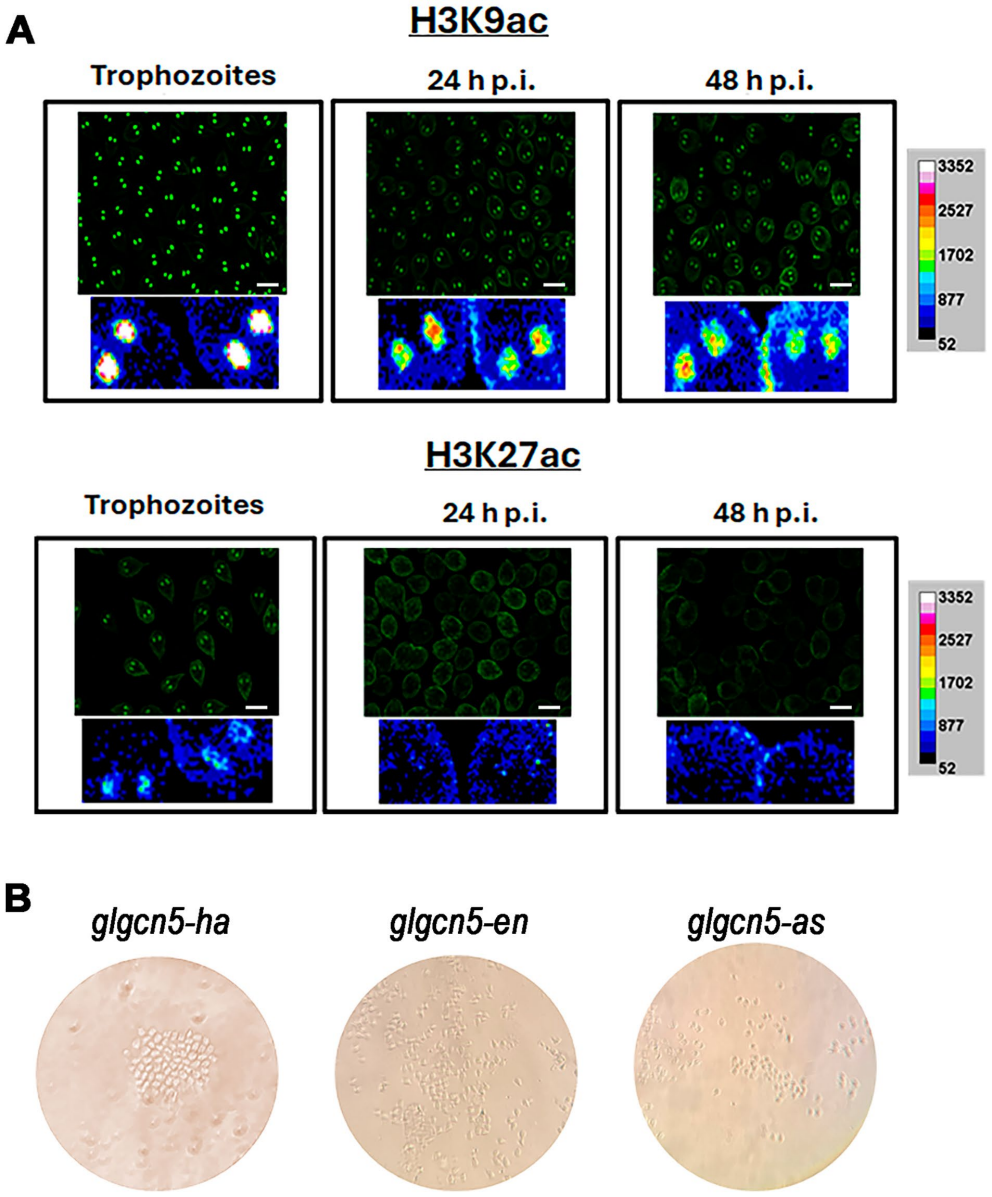
Localization and impact of histone acetylation on growth and encystation in *Giardia lamblia*. **(A)** Immunofluorescence analysis of histone H3K9 acetylation (H3K9ac) and H3K27 acetylation (H3K27ac) during trophozoite growth and encystation. Confocal microscopy images show the localization of H3K9ac in the nuclei of trophozoites, with strong marks detectable during growth that diminish but remain present at 48 h post-induction (h.p.i.). In contrast, H3K27ac marks are more discrete during growth and disappear after 24 h.p.i. Scale bars represent 10 μm. **(B)** Transfection results for GCN5 under different expression conditions in WB1267 strain trophozoites. Images depict transfection outcomes for *glgcn5-ha*, *glgcn5-en*, and *glgcn5-as* transgenic cells observed under a microscope. Despite successful transfections, cell proliferation was arrested in all conditions without forming a monolayer culture.

Based on previous studies by other authors indicating that clear histone deacetylation is necessary for encystation in the parasite, our first objective was to analyze the localization of histone acetylation marks during growth and encystation. We began by studying lysine 9 and 27 of histone H3, potential substrates of the KAT enzyme GCN5 in *G. lamblia*. Figure 1A shows immunofluorescence images analyzed via confocal microscopy using the anti-H3K9ac and anti-H3K27ac antibodies. Regarding H3K9ac, which is correlated with transcriptional activation, we found a strong mark localized in the nuclei during growth that diminished as encystation progressed. However, it was still present at 48 h p.i. On the other hand, the mark of H3K27ac, also involved in transcriptional activation, was more discrete in the nuclei during growth and disappeared after 24 h p.i. These results confirm previous findings and support that a general deacetylation is needed to allow encystation to progress.

### 3.2 Analysis of lysine acetyltransferases and their impact on parasite proliferation

In the genome *of Giardia lamblia*, there are five putative enzymes described that could act as KATs (Emery-Corbin et al., 2021). To start studying GCN5 (GL50830_10666), we designed primers to overexpress the enzyme under the tubulin promoter (glgcn5-ha transgenic cells) or the endogenous one (glgcn5-en transgenic cells) and to underexpress the expression we used the antisense strategy (glgcn5-as transgenic cells). After performing the transformations in bacteria, the plasmids were purified, the sequences were analyzed, and WB1267 strain trophozoites were transfected, adding puromycin for selection, as usual. Generally, if the cells are transfected 10 days after the addition of puromycin, the cultures begin to grow, forming a monolayer of trophozoites around day 14. We observed that the cells were transfected regardless of whether we performed the overexpression under the endogenous promoter or the tubulin promoter. Still, they remained in a latent state (Figure 1B). There were groups of cells that grew very slowly, but at no point did we detect cells moving or migrating, a usual behavior in trophozoite cultures. We repeated the procedure several times, changing the conditions of the cell/plasmid ratio and incubation times, obtaining similar results. The same occurred using the antisense strategy to reduce the expression of the enzymes. In none of the situations were we able to get a monolayer culture of *G. lamblia* that would allow us to continue the experiments.

These results prompted us to analyze two other KATs, named MYST1 (GL50803_17263) and MYST2 or ESA1 (GL50803_2851), whose putative substrates are present in histone H3 and histone H4 (Emery-Corbin et al., 2021). We repeated the procedure of designing primers for the three conditions, and after transfection, we found similar results to those with the enzyme GCN5, observing stalled replication. Therefore, overall, we conclude that histone acetyltransferases are indispensable for parasite proliferation *in vitro* and that any imbalance in enzyme expression induces an arrest in parasite proliferation.

### 3.3 Effects of garcinol on *Giardia lamblia* viability and morphology

**Figure 2:**
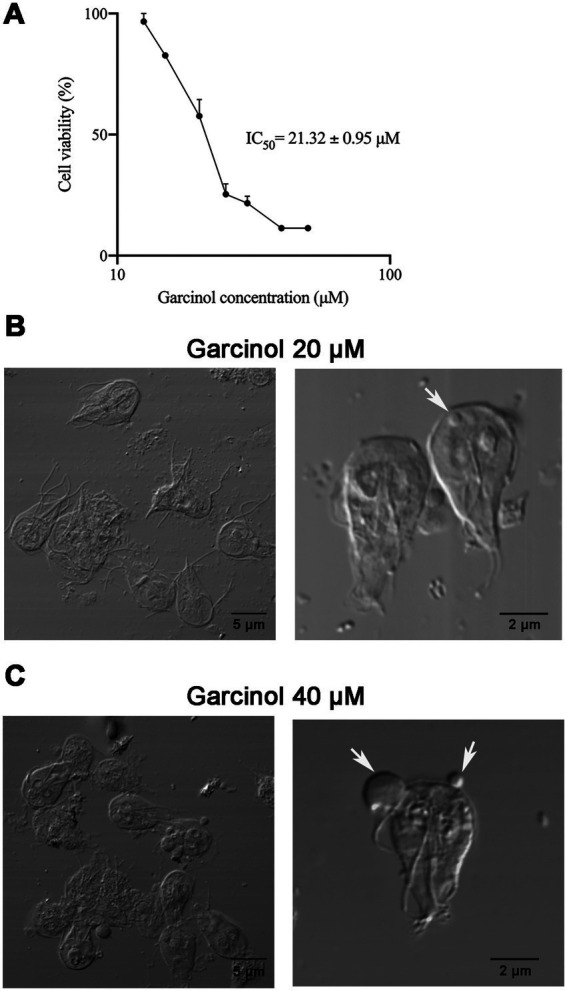
Effect of garcinol on the viability and morphology of *Giardia lamblia* trophozoites. **(A)** Cell viability assay using MTT to determine the IC_50_ of garcinol. Trophozoites were treated with increasing concentrations of garcinol (0–50 μm) for 48 h. The IC_50_ was determined to be 21.32 ± 0.95 μm. Cells incubated with the vehicle (DMSO) served as the control. **(B)** Differential interference contrast (DIC) images of trophozoites treated with 20 μm garcinol for 48 h. No significant changes in morphology were observed, although some cells exhibited round-shaped intracellular protrusions (white arrows). **(C)** DIC images of trophozoites treated with 40 μm garcinol for 48 h. At this higher concentration, trophozoites displayed clear signs of cellular deterioration, showed an increase in the size of intracellular protrusions, with a few extracellular structures visible (white arrows).

Enzyme inhibitors have been used to analyze the role of post-translational modifications of enzymes indirectly. Considering the essential role of acetylation for *G. lamblia* viability, we sought an acetylation inhibitor that could be used in *G. lamblia*. We selected a natural compound called garcinol, a polyisoprenylated benzophenone isolated from the dried rind of the edible fruit kokum (*Garcinia indica*), a plant native to southern India, which is a potent histone acetyltransferase inhibitor (Balasubramanyam et al., 2004). This compound has already been tested in other parasites, showing a decrease in global lysine acetylation in *T. gondii* and an arrest of parasite proliferation in both *T. gondii* and *P. falciparum* (Jeffers et al., 2016). We used concentrations of garcinol ranging from 0 to 100 μM, and we performed cell viability assays using the quantitative colorimetric technique with 3-(4,5-dimethylthiazol-2-yl)-2,5-diphenyltetrazolium bromide, or MTT. We determined that the IC_50_ of garcinol is 21.32 ± 0.95 μM. As a control, we always incubated the cells with the vehicle (DMSO) (Figure 2A). Based on these results, we decided to perform the following experiments during a 48 h treatment period using two different concentrations of garcinol: 20 μM (IC_50_) and 40 μM (2 × IC_50_) and DMSO in control cells, unless specified in a particular experiment.

Morphological analysis using DIC revealed that the cell morphology did not change significantly after treatment with 20 μM of garcinol. However, some trophozoites displayed round-shaped elevations inside the cells, with a few of these structures found in the extracellular space (Figure 2B). These features were more prominent with 40 μM of garcinol. The size of the protrusions increased considerably (Figure 2C). At this concentration, morphological changes indicative of cellular deterioration were observed.

**Figure 3:**
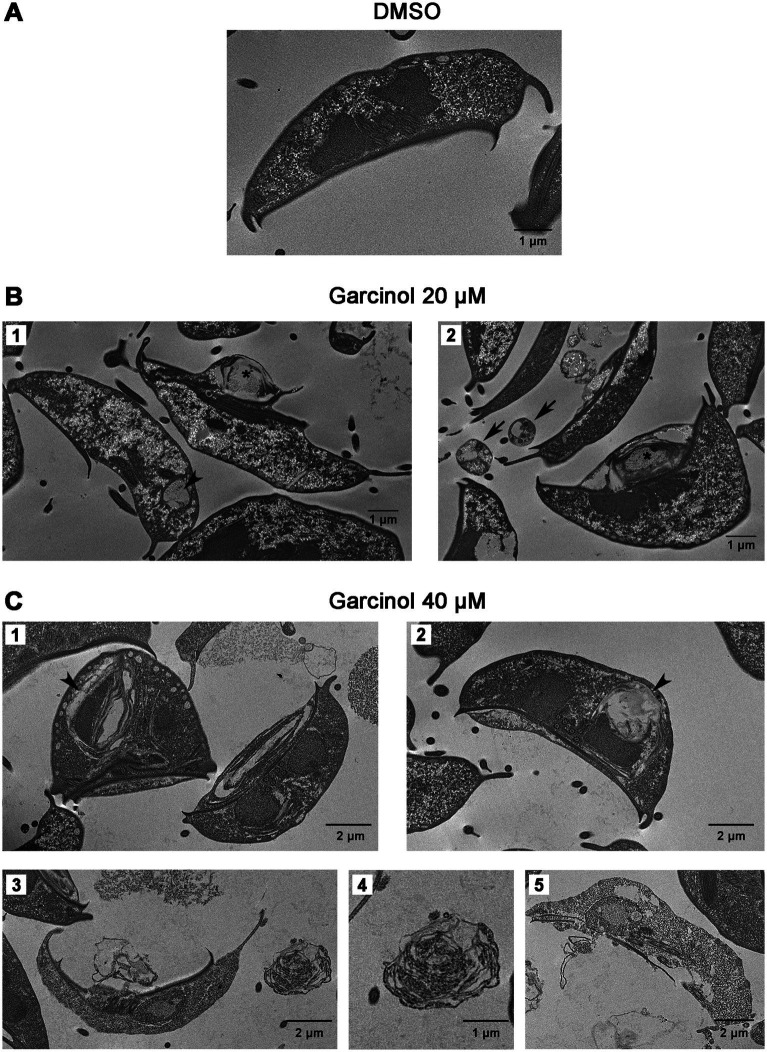
Ultrastructural changes in trophozoites following garcinol treatment observed via transmission electron microscopy. **(A)** Control trophozoites treated with DMSO exhibit normal morphology with no significant ultrastructural alterations. **(B)** Trophozoites treated with 20 μm garcinol display morphological changes, including ventral surface projections (asterisks) and the presence of lamellar structures in the cytoplasm (arrowhead). Apoptotic-like bodies containing cellular material were detected in the extracellular space (black arrows). **(C)** Trophozoites treated with 40 μm garcinol show more pronounced morphological alterations, including larger vacuoles containing lamellae (arrowheads). Cells also exhibit signs of cell death, such as membrane disruption and disorganization of the ventral disc and cytoplasmic structures (panels 3 and 5). In the extracellular medium, large granule-containing structures (panel 4) appear to hold remnants of cellular content. Scale bars indicate 1 μm or 2 μm, as specified.

Continuing our study, we delved into the ultrastructural elements of the cells using transmission electron microscopy. We observed projections on the ventral surface of trophozoites when treated with 20 μM garcinol (Figure 3B, asterisks). Additionally, in some trophozoites, lamellar structures were observed within the cytoplasm (arrowhead). In the extracellular space, we found apoptotic-like bodies containing cellular material (black arrows). When the cells were exposed to 40 μM garcinol, the morphological changes were more evident, with a greater number of vacuoles containing lamellae observed inside the cells, which also were larger in size (Figure 3C, arrowheads). No significant changes were observed in the peripheral vesicles or in the structure of the ventral disc, nor were any changes detected in chromatin condensation within the nuclei. In panels 3 and 5, cells show pronounced signs of cell death, such as disruption of the plasma membrane and the ventral disc, as well as disorganization of cytoplasmic structures. In the extracellular medium, large structures (4) that seem to contain granules were observed, which appear to hold remnants of cellular content.

### 3.4 Garcinol induces apoptosis-like death in *Giardia lamblia* trophozoites

**Figure 4:**
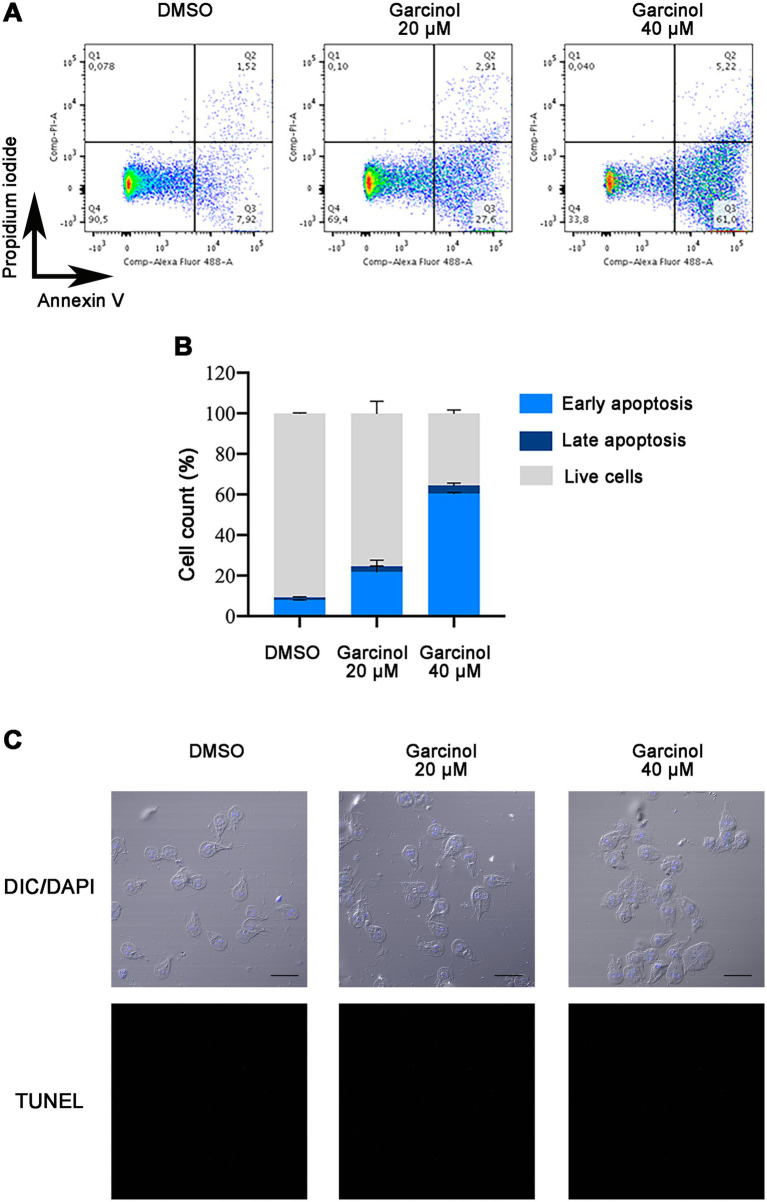
Analysis of apoptosis and necrosis in *Giardia lamblia* trophozoites treated with garcinol. **(A)** Representative scatter plots from flow cytometry analysis of *G. lamblia* trophozoites stained with Annexin V-Alexa 488 and propidium iodide (PI) after 24 h of treatment with DMSO (control), 20 μm garcinol, or 40 μm garcinol. The quadrants represent: Q1 (Annexin V−/PI+): necrotic cells; Q2 (Annexin V+/PI+): late apoptotic/necrotic cells; Q3 (Annexin V+/PI−): early apoptotic cells; and Q4 (Annexin V−/PI−): live cells. **(B)** Bar graph showing the percentage distribution of live, early apoptotic, late apoptotic, and necrotic *G. lamblia* trophozoites under each treatment condition as measured by flow cytometry. Treatment with increasing concentrations of garcinol resulted in a dose-dependent increase in early apoptosis, reaching nearly 60% at the highest concentration tested (40 μm). A concurrent increase in late apoptosis/necrosis was also observed, though it comprised only about 5% at the highest concentration. **(C)** DNA fragmentation was analyzed using TUNEL assays on treated and control cells and observed using confocal microscopy. DAPI was used to visualize nuclei. No TUNEL-positive cells were observed. Scale bar: 10 μm.

It was previously shown that the histone acetyltransferase inhibitor garcinol induces apoptosis and DNA fragmentation in HeLa cells at 30, 70, and 100 μM (Balasubramanyam et al., 2004). To analyze whether *G. lamblia trophozoites* suffered apoptosis/necrosis under garcinol treatment, we performed flow cytometry assays using PI, a membrane-impermeable nucleic acid-specific dye, that reflect cell death (late apoptosis/necrosis), and Annexin V that detect phosphatidylserine when it is exposed to the plasma membrane of the cells during early apoptosis (Figure 4A). After 24 h of treatment, we observed that the percentage of cells in early apoptosis rose in correlation with the increasing concentration of garcinol, with almost 60% of cells in early apoptosis under 40 μM of garcinol. No differences were observed in the proportion of necrotic cells under treatment (Q1). Also, cells in late apoptosis/necrosis increase, with 5% of cells in this stage under the highest concentration of garcinol (Figure 4B).

During apoptosis, biochemical and morphological events lead to programmed cell death. One of these events is the activation of specific endonucleases, which are enzymes that cut DNA into fragments from 50 to 300 kb and are further degraded to low molecular weight oligonucleoside-sized fragments (Di Filippo and Bernardi, 2009). The TUNEL assay was performed on cells treated with DMSO, garcinol 20 μM, and 40 μM; however, no TUNEL-positive cells were found under any condition (Figure 4C). Since the entire apoptotic machinery seems absent in *Giardia*, garcinol probably induces apoptotic-like cell death through a non-classical pathway.

### 3.5 Production of reactive oxygen species in *Giardia lamblia* trophozoites after garcinol treatment

**Figure 5:**
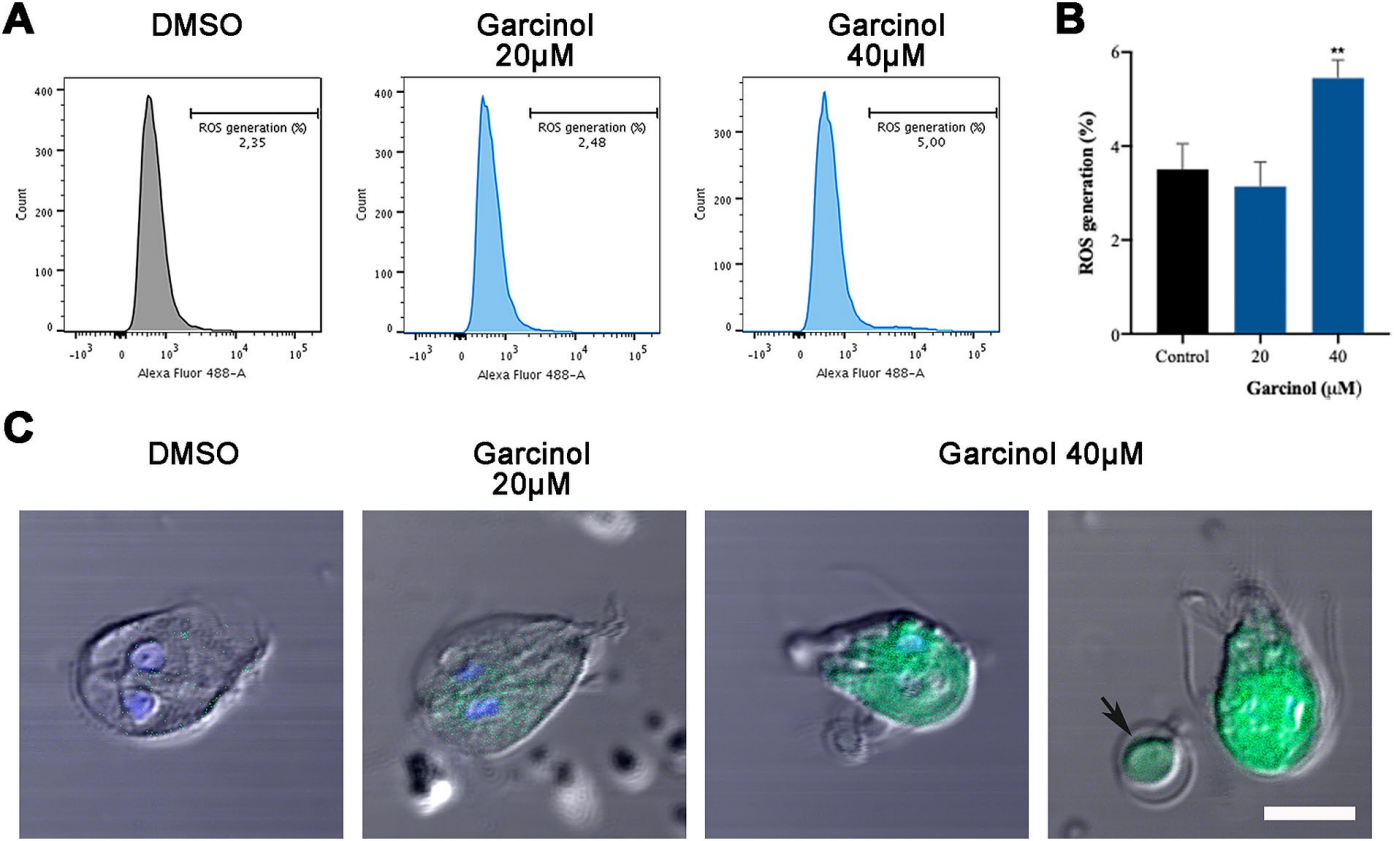
Production of ROS in *Giardia lamblia* trophozoites after garcinol treatment. **(A)** Flow cytometry analysis of reactive oxygen species in control and garcinol-treated *G. lamblia* trophozoites. No significant differences were observed in the percentage of ROS-positive cells between the control group and those treated with 20 μm garcinol. **(B)** Flow cytometry data showing a doubling of ROS-positive cells in trophozoites treated with 40 μm garcinol compared to the control group, (**p < 0.01). **(C)** Immunofluorescence assay illustrating the distribution of ROS within the cytoplasm of *G. lamblia* trophozoites. The presence and intensity of ROS increase with higher concentrations of garcinol. Black arrows indicate ROS presence in apoptotic-like bodies, suggesting cytoplasmic fragmentation leading to apoptosis. Scale bar: 5 μm.

To delve deep into the mechanism that finally induces cell death in *G. lamblia* under garcinol treatment, reactive oxygen species (ROS) were analyzed using flow cytometry and immunofluorescence assays. We found no differences in the percentage of ROS generation between control and trophozoites treated with 20 μM of garcinol (Figures 5A,B). At 40 μM, the rate of positive cells doubled compared to the control. Through fluorescence assays, we analyzed the ROS-positive cells. Considering that this assay is performed on live cells, in Figure 5C we show examples of ROS-positive cells, even though the majority were negative. We observed that as the garcinol dose increased, the distribution of ROS in the cell cytoplasm also increased. Additionally, the presence of ROS can be observed in apoptotic-like bodies (black arrow). After a thorough analysis of these results, we can conclude that ROS production is not the primary mechanism by which garcinol induces cell death, considering the low percentage of ROS-positive cells, but the presence of ROS in the round-shape structures reinforces the idea that there is a fragmentation of the cytoplasm of the cells, that finally produces apoptotic-like bodies.

### 3.6 The cytoskeleton of *Giardia lamblia* is affected by garcinol

**Figure 6:**
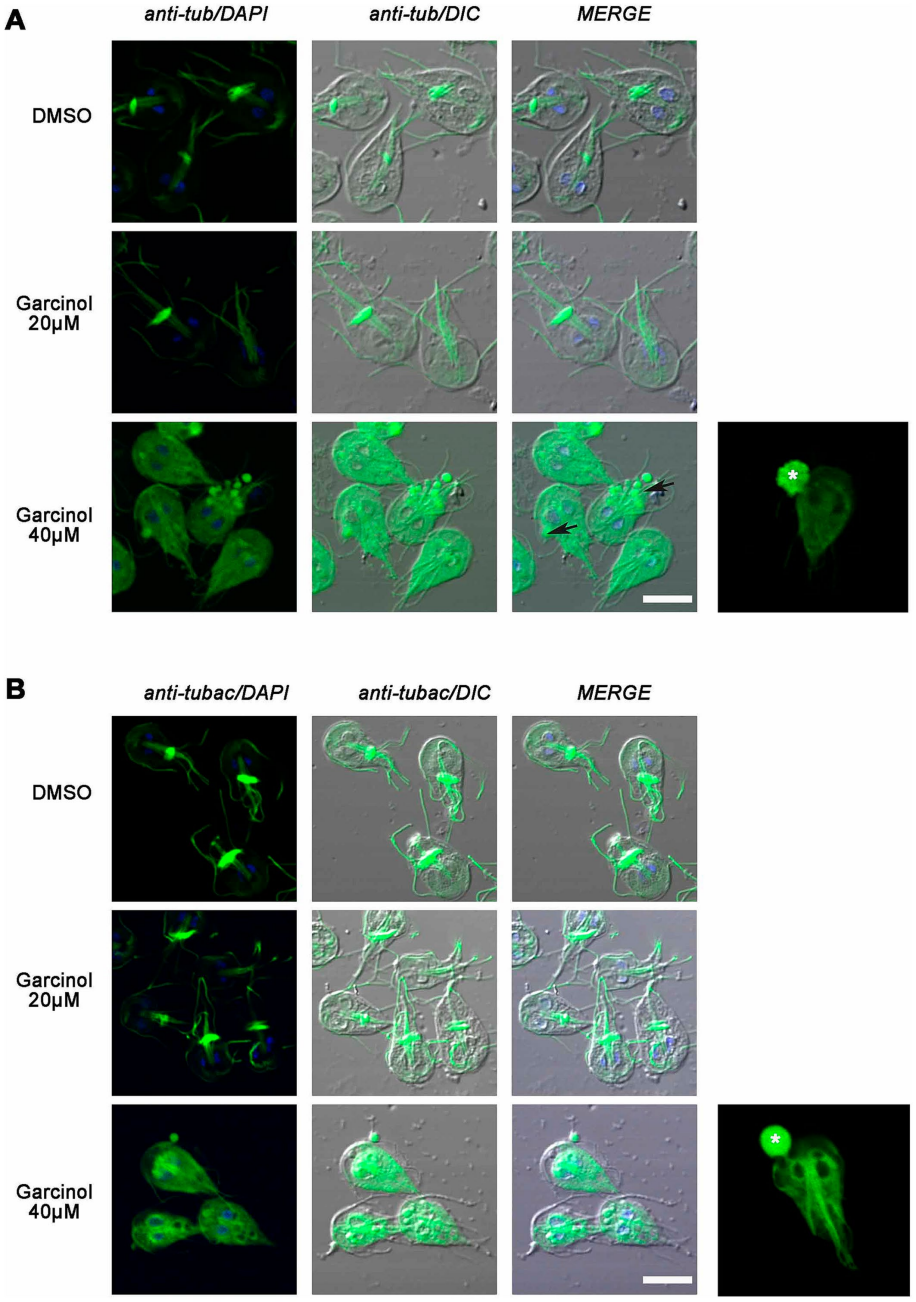
Analysis of the localization of tubulin and acetylated tubulin in *Giardia lamblia* trophozoites treated with garcinol. **(A)** Treatment with 20 μm garcinol resulted in tubulin localization primarily in the flagella, with no staining observed in the median body. At 40 μm garcinol, tubulin was distributed throughout the cytoplasm, with some retention in the flagella, while the median body remained unstained. Large vesicles or membrane blebs (asterisk) were marked with tubulin. **(B)** Acetylated tubulin showed similar localization patterns, with a loss of median body marking at lower concentrations and a cytoplasmic distribution at higher concentrations. Large vesicles or membrane blebs containing acetylated tubulin were also observed (asterisk). Scale bars: 5 μm.

Having observed the morphological changes, we explored whether garcinol also affected the localization of tubulin and acetylated tubulin in trophozoites treated using fluorescence microscopy. We found that the classical localization of tubulin in the median body and flagella changed after garcinol treatment (Figure 6A). When the cells were treated with 20 μM garcinol, we observed that in most cells, tubulin remained localized in the flagella. However, no staining was observed in the median body, which was also not evident in the DIC images. When the cells were treated with 40 μM garcinol, tubulin was observed distributed throughout the cytoplasm, with some staining retained in the flagella. No localization in the median body was observed in any of the cells, and the large vesicles or membrane blebs, both localized intracellular and extracellular (asterisk), were marked with tubulin.

Like many protozoan parasites, most of the tubulin in *G. lamblia* is acetylated, showing flagella, median body, and parts of the ventral disc marked with the antibody. We observed similarities to what was seen with tubulin, with a loss of marking in the median body at lower garcinol concentrations and a clear cytoplasmic distribution at higher concentrations (Figure 6B). Again, we observed those large vesicles or membrane blebs, which contained acetylated tubulin inside or emerging from the cell (asterisk). Overall, there seems to be a redistribution of tubulin, which may be associated with profound changes in the parasite’s ultrastructure but no decrease in tubulin acetylation.

### 3.7 Garcinol affects histone acetylation during growth

**Figure 7:**
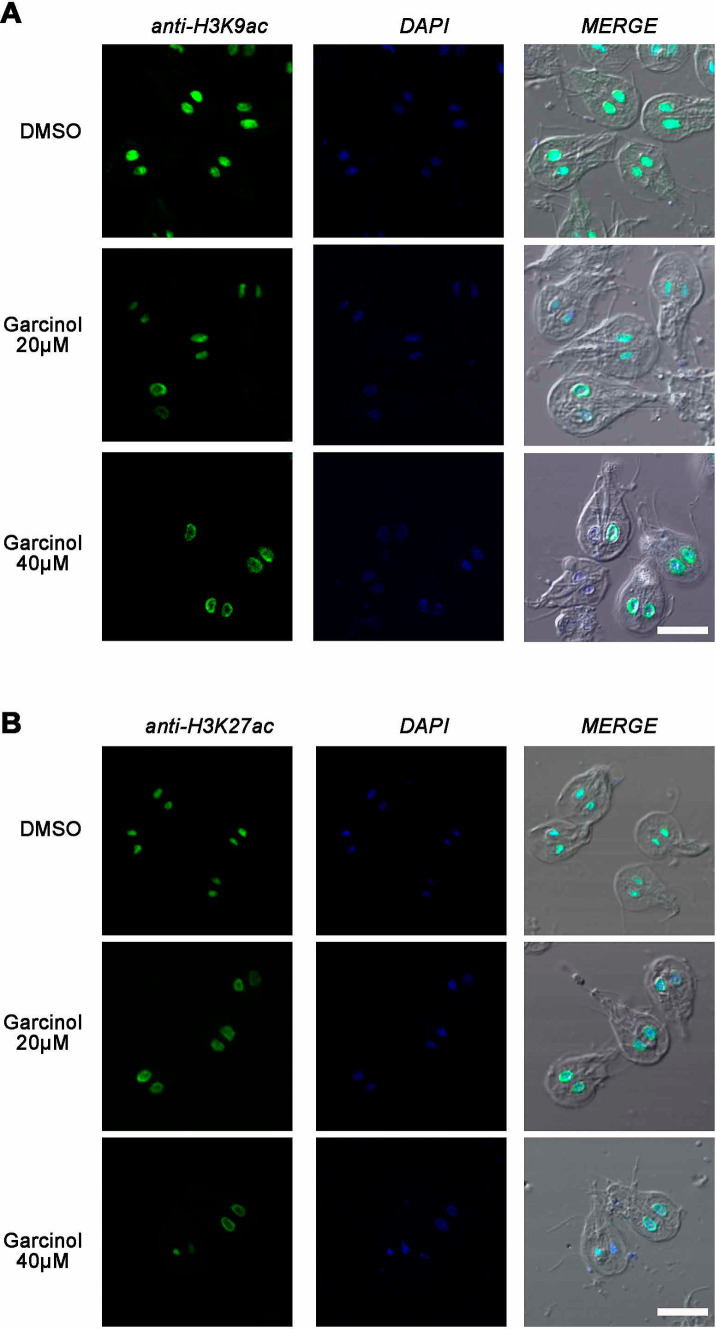
Garcinol affects histone acetylation during growth. Immunofluorescence assays and confocal microscopy were performed to analyze the effects of garcinol on the acetylation of lysine residues 9 and 27 on histone H3 in WB1267 cells treated with 20 μm and 40 μm Garcinol for 48 h. **(A)** Treatment with DMSO served as a control, showing distinct nuclear localization of the acetylated lysine 9 mark (anti-H3K9ac). At 20 μm garcinol, a reduction in fluorescence intensity was observed within the nuclei, with some cells exhibiting a perinuclear localization of the mark. At 40 μm Garcinol, the acetylation mark was predominantly perinuclear, with some cells lacking the H3K9ac mark entirely. **(B)** For lysine 27 (anti-H3K27ac), DMSO treatment showed discrete nuclear localization. Following treatment with 20 μm garcinol, the nuclear localization was lost, and a perinuclear pattern emerged, which became more pronounced at 40 μm garcinol, with some cells showing minimal marking. Scale bars: 5 μm.

Garcinol is a potent inhibitor of histone acetyltransferases p300 and PCAF. It also inhibits histone acetylation *in vivo* but does not affect the deacetylation of histones (Balasubramanyam et al., 2004). To analyze whether changes in acetylation of lysine residues 9 and 27 on histone H3 were observed, we incubated WB1267 cells in the presence of garcinol at 20 μM and 40 μM for 48 h. Following the treatment, we performed immunofluorescence assays and confocal microscopy. Regarding the acetylated lysine 9 mark on histone H3, we observed that even at the IC_50_ concentration, there was a change in the acetylation pattern, showing reduced fluorescence intensity within the nuclei. The acetylation mark was sometimes observed in a perinuclear location (Figure 7A). The effect was more evident when treating the cells with 40 μM garcinol, where we no longer observed the mark within the nuclei but rather at the periphery. Notably, in some cells, the mark was present in only one of the nuclei. Finally, we found cells without the H3K9ac mark within the nuclei.

In cells treated with DMSO, the acetylation mark of lysine 27 on histone H3 was similar to that observed in untreated trophozoites, where the nuclear mark was discrete (Figure 7B). Upon treatment with 20 μM garcinol, the nuclear localization was lost and a clear perinuclear pattern was evident, in both or only one of the nuclei. The mark is barely observable at 40 μM garcinol treatment and in some trophozoites. These results indicate that garcinol profoundly affects the acetylation of both lysine 9 and lysine 27 on histone H3 but differentially compared to a physiological situation such as encystation.

### 3.8 Evaluating *Giardia lamblia* recrudescence after drug treatment

**Figure 8:**
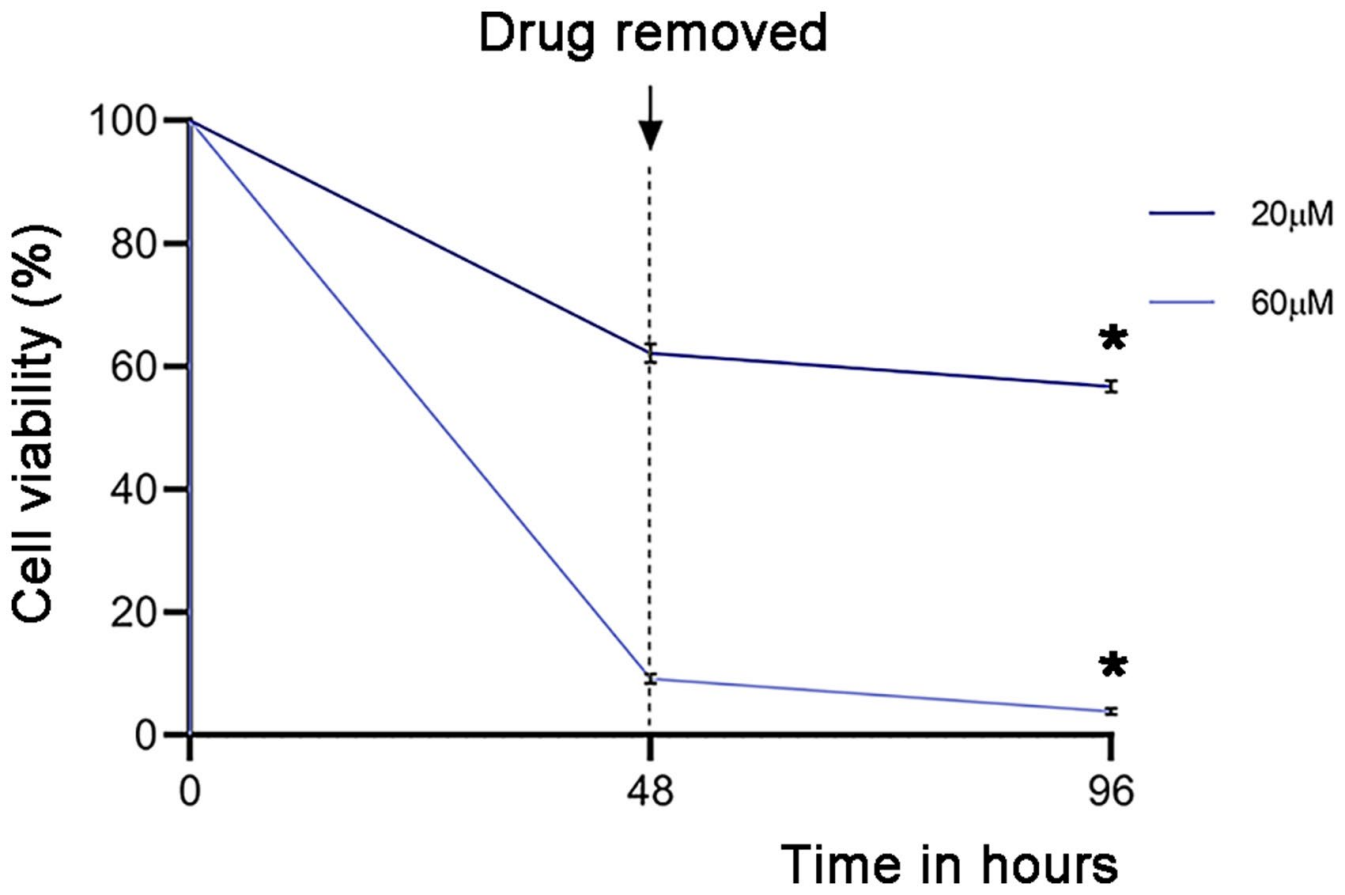
Analysis of the activity of garcinol in blocking recrudescence of *Giardia lamblia*. Parasite viability was measured using MTT assays after treatment with IC_50_ (20 μm) and IC_90_ (60 μm) concentrations, followed by drug removal for 48 h. The graph depicts the percentage of the signal compared to DMSO treatment (100%). Significant differences were determined by a paired one-tailed Student’s t-test (*p < 0.05; **p < 0.01).

Having observed the profound effect of garcinol on *Giardia lamblia* viability, we analyzed if garcinol induced a giardiacidal versus giardiastatic effect. Therefore, recrudescence was evaluated by testing the parasite’s viability when treated with the compounds IC_50_ (20 μM) and IC_90_ (60 μM) before and after 48 h of drug removal to determine if the surviving cells could proliferate in the absence of the drug. (Figure 8). Recrudescence was defined as viability above 10% post-recovery, significantly different (*p* < 0.05) from viability observed after 48 h treatment with garcinol alone. We found that garcinol effectively prevented recrudescence, suggesting a giardiacidal effect ideal for treating parasitic diseases and preventing recurrent infections caused by dormant parasites. This result highlights the potential of garcinol as a promising drug for the treatment of giardiasis.

## 4 Discussion

In the present study, we explore the critical role of protein acetylation in *G. lamblia*, demonstrating its significance as a post-translational modification essential for the parasite’s survival. Lysine, a basic amino acid with a positively charged amino group under physiological conditions, undergoes a transformation when an acetyl group is added, neutralizing this charge. This modification alters the properties of lysine and, consequently, the function of the protein. Lysine acetylation occurs in both histone and non-histone proteins, exerting profound effects on protein function, regulation, and overall cellular processes (Gil et al., 2017; Vandrisse and Escalante-Semerena, 2019). In eukaryotic cells, the repertoire of acetylated proteins has increased substantially. This has revealed the diversity of target proteins for this modification, allowing researchers to propose that ‘acetylation rivals phosphorylation,’ a well-known modification involved in various regulatory pathways. This prediction is valid year after year and is no different for protozoan parasites.

Although this modification is extensively studied in other parasites, in *G. lamblia*, most studies address the counterpart of acetylation, which is deacetylation (Sonda et al., 2010; Carranza et al., 2016; Wang et al., 2016; Herrera et al., 2019; Lagunas-Rangel and Bermúdez-Cruz, 2019; Emery-Corbin et al., 2021). Only the localization of some acetylated residues in histones has been reported through immunofluorescence assays, as well as the presence of these modifications using immunoblotting (Sonda et al., 2010; Carranza et al., 2016; Emery-Corbin et al., 2021). The present study analyzed the localization kinetics of two activating histone marks: lysine 9 and lysine 27 in histone H3. The acetylation of lysine 9 was reported through mass spectrometry studies and immunoblot assays, showing a strong mark on histone H3 in the latter (Emery-Corbin et al., 2021). Regarding the immunofluorescence assays, both in the study published by Carranza and in ours, we found acetylation marking of lysine 9, although with different intensities, due to the use of different commercial antibodies. However, the nuclear localization indicates clear specificity of the antibodies (Carranza et al., 2016). During encystation, we confirmed that there was deacetylation of both marks. This result was previously observed using an antibody against acetylated lysine (AcK) but not against residues in histones (Sonda et al., 2010). This deacetylation of histones during encystation likely indicates that other post-translational modifications, such as methylation, target these residues. Mass spectrometry assays performed on growing trophozoites described mono-, di-, and tri-methylation of lysine 9 and di-methylation of lysine 27. These marks are associated with transcriptional silencing and heterochromatin formation. Therefore, in our laboratory, we are studying these methylation marks during encystation, not only by immunofluorescence but also by conducting ChIP-seq assays to analyze which genes are associated with these modifications. Considering that during encystation, some genes must be upregulated to develop the process, while others must be downregulated to enter the latency period, the analysis of PTMs and associated genes could contribute to understanding this process.

Regarding KAT enzymes, we found that the enzymes that we studied are essential for the parasite’s survival. Both overexpression with a strong promoter such as tubulin, expression from their endogenous promoter, and silencing through antisense resulted in trophozoites remaining in a latent state. Similar results were observed in other parasites as well. For example, in *T. gondii*, the histone acetyltransferase TgGCN5-A, the counterpart of KDAC, plays a crucial role in stress-induced bradyzoite differentiation, and it is dispensable for parasite proliferation under standard culture conditions (Bhatti et al., 2006). Other histone acetyltransferases, such as TgGCN5-B, TgMYST-A, and TgMYST-B, are essential for parasite viability, as disruption of the loci encoding these enzymes does not produce viable parasites (Dixon et al., 2010). However, overexpression of a dominant-negative form of TgGCN5-B with a point mutation that abolishes enzymatic activity led to a rapid arrest in parasite replication, accompanied by decreased histone H3 acetylation at lysine residues and reduced expression of TgGCN5-B target genes in the transfected parasites (Wang et al., 2014). Additionally, parasites do not tolerate overexpression of TgMYST-A, which is lethal, but mutating the HAT domain can reverse this effect in overexpressing parasites. Moreover, overexpression of TgMYST-B results in a slow-growth phenotype but mutating the HAT domain renders transgenic parasites remarkably resistant to DNA damage induced by alkylating agents (Smith et al., 2005; Vonlaufen et al., 2010). Future experiments in our laboratory involve the identification of the active domain through bioinformatic analysis and the subsequent overexpression of a dominant-negative form of KATs with a point mutation to abolish enzymatic activity. Another possibility is the production of recombinant proteins and monoclonal antibodies to analyze their localization and purification through immunoprecipitation assays. We believe that through these experiments, we can advance the study of these enzymes that are critical for the survival of *G. lamblia*.

Furthermore, although many human proteins contain acetyl-lysine, only a dozen of these enzymes have been identified, posing the enigma of how such a small number of enzymes maintain such a large acetylome. In this context, molecular characterization indicates that some of these enzymes are promiscuous and target multiple protein substrates in addition to histones. In *G. lamblia*, a recent study identified 2,999 acetylation sites on 956 proteins, representing the first acetylation proteome for this organism (Zhu et al., 2021). The acetylated proteins were enriched in processes related to peptide and amide biosynthesis and metabolism, suggesting amino acids play a significant role in its energy metabolism and metabolic conversion. Considering this result and ours, we may infer that the critical role of acetylation in histone and non-histone proteins makes this PTM crucial for the survival of *G. lamblia*. Therefore, we explored acetylation inhibitors as potential therapeutic targets. Various approaches have been employed to identify HAT inhibitors, including constructing HAT substrate mimics, research on natural products, and high-throughput and virtual screening [reviewed in Maran et al. (2021)]. We used garcinol to evaluate its antigiardial activity and potential influence on protein acetylation. This natural product has demonstrated anti-inflammatory and antioxidant activity, as well as antiproliferative, anti-angiogenic, and proapoptotic activities, in addition to KAT inhibition (Semwal et al., 2015). These characteristics have led to testing this natural product in various types of cancer (Saadat and Gupta, 2012). Related to parasites, a study explored the effect of garcinol on the replication of *Toxoplasma gondii* and *Plasmodium falciparum* (Jeffers et al., 2016). These authors found that this compound inhibits *Toxoplasma* tachyzoite replication and *P. falciparum* erythrocytic asexual replication. Moreover, they found that garcinol inhibits TgGCN5b, reduces lysine acetylation levels, and reduces autoacetylation of TgGCN5b. Although it seemed to be a promising drug for the treatment of these parasites, *in vivo* trials on animals infected and treated with garcinol showed that garcinol (10 or 20 mg/kg) did not protect against acute infection, likely due to high-affinity albumin binding limiting tissue distribution.

Related to the effects of garcinol on *G. lamblia* trophozoites, we found deacetylation of lysines 9 and 27 of histone H3. However, when we compared these marks with what happens during encystation, which is a physiological and regulated process, we observed that in the case of lysine 9, under treatment with a concentration of 20 μM of garcinol, several nuclei showed an absence of the mark inside. At double the IC_50_, some cells showed no mark or only in one of their nuclei, whereas during encystation, what decreases is the intensity of fluorescence within the nuclei. Regarding lysine 27, the mark disappears from the nuclei during encystation, while with garcinol treatment, a pattern similar to that observed for lysine 9 is seen. These differences might be due to the activation of KDACs during encystation, which deacetylates these residues. In contrast, as demonstrated in other models, garcinol possibly affects lysine acetylation either directly or indirectly. Since we could not transfect the KATs, we cannot delve deeper into these studies yet, which will be the subject of future experiments.

The cytoskeleton of *G. lamblia* is composed of microtubules, which are organized into different structures, such as the median body, the ventral disc, the funis, and the flagella. These structures are involved in critical processes for the survival of trophozoites, such as adhesion, cell division, and movement. Therefore, compounds that affect the cytoskeleton are crucial for treating giardiasis. Garcinol causes a clear redistribution of tubulin and its acetylated form, which can be observed in the cell’s cytosol, likely due to microtubule depolymerization. Similar results were observed under treatment with nocodazole (Mariante et al., 2005). While our study primarily focused on giardial KATs, it is possible that other enzymes, not necessarily from this family, could also contribute to tubulin redistribution. Regarding garcinol, although it is known to primarily target KATs, it could potentially interact with other enzymes non-specifically or it might affect an enzyme critical for tubulin organization. As the concentration of garcinol increases, deposits of tubulin can be observed within the cytoplasm, and in some cases, plasma membrane blebs are observed. These latter structures have been associated to apoptotic-like bodies generated in cells undergoing programmed cell death. This appears to be the case under treatment with garcinol, as we observed using flow cytometry, phosphatidylserine exposure on the plasma membrane, which occurs in the early stages of apoptosis. Additionally, we observed a dose-dependent increase in both the size and number of apoptotic-like bodies as the drug concentration rose. We found tubulin, acetylated tubulin, ROS, and an absence of acetylated histones within these bodies. In *G. lamblia*, the process of apoptosis is not fully described, and studies indicate that it lacks caspases, suggesting that programmed cell death occurs through mechanisms different from those in other eukaryotic cells (Mariante et al., 2005; Bruchhaus et al., 2007; Benchimol et al., 2023). Moreover, it was observed that not all compounds inducing apoptosis exhibit the same characteristics or results. For example, we observed ROS production in a minority population, indicating that although ROS production is characteristic of classical apoptosis, this does not occur under garcinol treatment.

Finally, in the recrudescence assays, the trophozoites could not replicate and recover after the drug was removed for 48 h, even at the IC_50_ and especially at the 2 × IC_50_. This is crucial when considering a drug for treating parasites with a high replication rate. Recrudescence is regarded as a sign of treatment failure, needing a shift to more potent drugs or drug combinations (Looareesuwan et al., 1992). Drug treatment selects resistant parasite strains that multiply (Spencer et al., 1984; Thaithong et al., 1988). It is crucial to highlight that the first-line treatment for this parasitosis, based on members of the 5-nitroimidazole family such as metronidazole (MTZ) and tinidazole, fails in up to 20% of cases and may contribute to the development of resistance to the main giardicidal drugs, as well as cross-resistance between different drugs, as seen with MTZ and nitazoxanide (Ansell et al., 2015; Emery-Corbin et al., 2021; Loderstädt and Frickmann, 2021). Therefore, identifying potential new drugs that prevent recrudescence may be the key to finally combating giardiasis.

Our study highlights the significance of protein acetylation in the survival of *G. lamblia*, emphasizing its role as a critical post-translational modification. We observed that garcinol affects tubulin distribution, leading to apoptosis-like characteristics, further validating its potential as an anti-parasitic agent. These findings, coupled with the inability of trophozoites to recover after drug removal, suggest that garcinol is a promising candidate for treating parasites with high replication rates.

## Data Availability

The datasets presented in this study can be found in online repositories. The names of the repository/repositories and accession number(s) can be found in the article/supplementary material.

## Footnotes

^1^ giardiadb.org

^2^ http://fiji.sc/wiki/index.php/Fiji
